# Direct-write polymer nanolithography in ultra-high vacuum

**DOI:** 10.3762/bjnano.3.6

**Published:** 2012-01-19

**Authors:** Woo-Kyung Lee, Minchul Yang, Arnaldo R Laracuente, William P King, Lloyd J Whitman, Paul E Sheehan

**Affiliations:** 1 Chemistry Division, U.S. Naval Research Laboratory, Washington, DC 20375, USA; 2 US Patent and Trademark Office, Alexandria, VA 22314, USA; 3 Department of Mechanical Science and Engineering, University of Illinois Urbana-Champaign, Urbana, IL 61801, USA; 4 Center for Nanoscale Science and Technology, National Institute for Science and Technology, Gaithersburg, MD 20899, USA

**Keywords:** additive lithography, polymer, scanning probe lithography, ultra high vacuum

## Abstract

Polymer nanostructures were directly written onto substrates in ultra-high vacuum. The polymer ink was coated onto atomic force microscope (AFM) probes that could be heated to control the ink viscosity. Then, the ink-coated probes were placed into an ultra-high vacuum (UHV) AFM and used to write polymer nanostructures on surfaces, including surfaces cleaned in UHV. Controlling the writing speed of the tip enabled the control over the number of monolayers of the polymer ink deposited on the surface from a single to tens of monolayers, with higher writing speeds generating thinner polymer nanostructures. Deposition onto silicon oxide-terminated substrates led to polymer chains standing upright on the surface, whereas deposition onto vacuum reconstructed silicon yielded polymer chains aligned along the surface.

## Introduction

The deposition of materials in vacuum is the foundational technology for creating modern electronic circuits; a vacuum being essential both to preserve the cleanliness of the substrate and the deposited materials and to minimize the creation of defects [1]. Consequently, most deposition techniques from thermal evaporation to atomic layer deposition require a high level of vacuum, preferably ultra-high vacuum (UHV), to be used effectively. While the suite of established vacuum deposition technologies is vast and capable of highly precise deposition, there are relatively few methods to perform additive lithography in a single deposition step. Additive lithography deposits only the material that is needed for the intended device in the correct position. This is in contrast to the standard practice where an entire film is generated, the great majority of this film is then removed. In addition to the benefit of reduced material cost, additive techniques have further benefits, including the ability to create softer, heterogeneous structures – such as polymers – that would be contaminated or destroyed by the multiple requisite coating and removal steps associated with conventional “lift-off” lithography. To date, additive lithographies such as inkjet [2], dip-pen nanolithography (DPN) [3] and micro-contact printing [4] have been limited to deposition under ambient pressures, and therefore cannot achieve the benefits of the controlled environment under vacuum.

**Figure 1:**
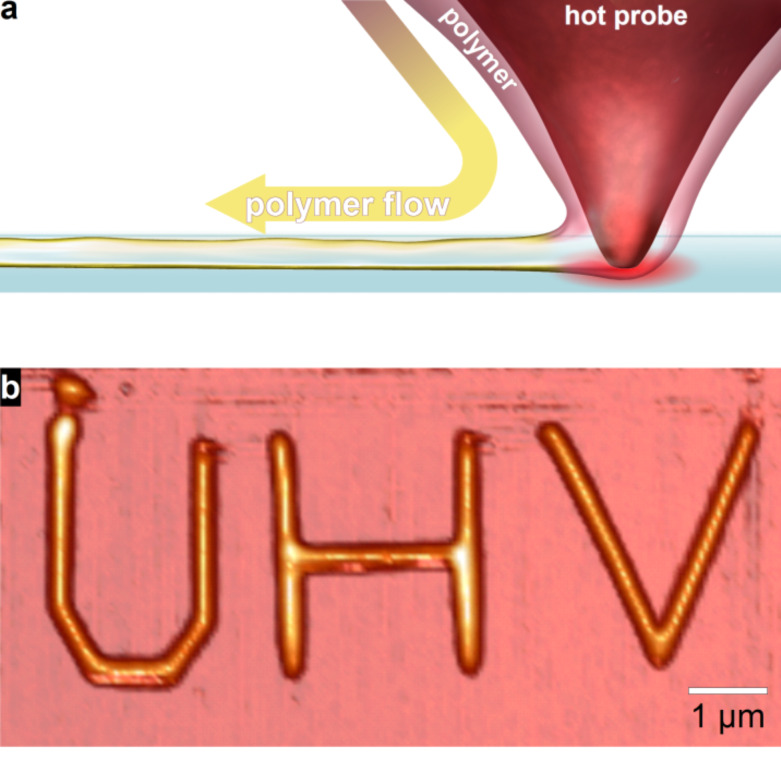
(a) Schematic of the tDPN process which uses a heated scanning probe microscope tip to deposit polymer from a moving tip. (b) Leaving the tip in contact, deposition is started and stopped by turning the heat on and off as shown by writing “UHV”. The poly(3-dodecylthiophene) (PDDT) was written on SiO_2_ (non-UHV prepared) in UHV (~10^−10^ Torr). The height of each polymer line was 20 nm (= 8 ML) while the polymer width was 150 nm (fwhm).

One type of additive lithography is scanning probe lithography (SPL) where sharp probes either guide the deposition of material to a substrate or modify previously deposited films [5–6]. In the case of DPN, the AFM probe can be used to write a wide range of molecular inks with resolutions down to 15 nm [3,7–8]. However, in conventional DPN writing depends on the intrinsic fluidity of the ink molecules or on the creation of ink fluidity using solvents [9]. Unfortunately, inks and solvents that have sufficient intrinsic fluidity for DPN evaporate quickly in vacuum. This paper reports that thermal dip-pen nanolithography (tDPN) [10] can deposit polymer nanostructures from a heated AFM tip in a high vacuum environment (Figure 1b). In tDPN, the probe temperature may be varied precisely within microseconds over a temperature range of 1000 °C. The probe temperature controls the viscosity of the coated ink allowing independent control over the overall deposition rate and the ability to turn off and on deposition (Figure 1a). Many different materials (e. g., metals [11], nanoparticles [12], and SAM molecules [10]) have been deposited using this technique. Thermal DPN closely mirrors the capabilities of conventional DPN but with greater control over the ink flow [5]. Critically, the heat from the probes enables the deposition of high melting point inks such as polymers that also have low volatility and so may be deposited under a vacuum.

## Results and Discussion

Our initial approach for depositing organic inks was to attempt DPN with octadecanethiol (ODT), a classic ink for DPN that reproducibly transfers to the substrate. However, it was found that the ink on the DPN tip would invariably evaporate in the load lock chamber (~10^−7^ Torr) leaving insufficient coverage for observable deposition. Evaporation is readily observed visually since the ink leaves a haze on the tip that is absent after placing in a load lock chamber. This anecdotal observation was more rigorously examined by creating a sample that mimicked the DPN tip surface chemistry: A silicon oxide on a silicon chip that was coated by holding it over ODT in a scintillation vial heated to 65 °C, for 30 min. This procedure produced an ODT film that was 20 nm thick (measured by ellipsometry). After placing the chip briefly under vacuum in a load lock chamber (~10^−7^ Torr), no ODT film was detectable. Additional attempts with less volatile inks – such as eicosanethiol – yielded similar results, leading us to conclude that typical inks used in conventional DPN cannot be used for DPN under vacuum.

While alkanethiols could not be deposited, we found that heated probes would retain and deposit polymer in UHV. For this work, we chose the polymer to be poly(3-dodecylthiophene) (PDDT), a conducting polymer that has found widespread usage in organic electronics (Figure 1b) [13]. PDDT is also interesting because it becomes highly ordered, forming self-assembled layers on a silicon surface [14], when it is properly annealed. This ordering increases its ability to conduct current after electron beam exposure [15].

The probe temperature was controlled by applying current through the probe heater [16]. One of the advantages of UHV tDPN is the lower melting point of inks under UHV. Because the molar volume of PDDT is lower in solid form than in liquid form, thermodynamics indicate that its melting point should drop as the surrounding pressure is lowered. Thus, while PDDT routinely deposits at its melting point of 120 °C in air, we observed that the writing temperature of PDDT could be decreased down to ~100 °C in UHV. As a result, the temperature window between melting and thermal decomposition of PDDT (175 °C in air) widens, thereby enabling greater control of line widths and thicknesses deposited in UHV. The lower deposition temperature also reduces the risk of thermal damage when applied to pre-fabricated devices.

**Figure 2:**
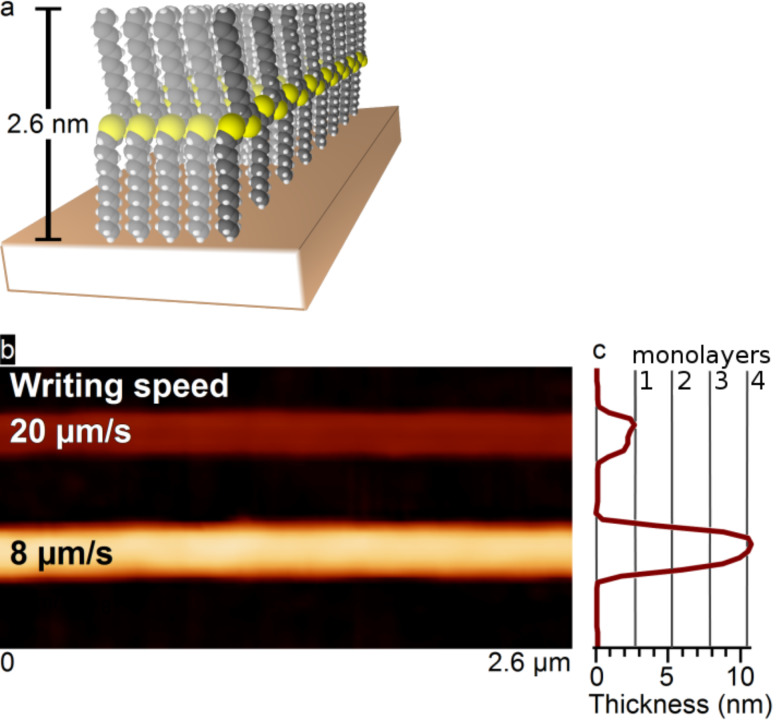
Orientations of UHV deposited polymer. (a) PDDT typically organizes in such way that the polymer is oriented normal to the surface with a monolayer height of 2.6 nm. (b) Deposition of polymer at different speeds on a non-UHV prepared substrate showing the upright orientation in (a). By varying the tip speed, the scanning probe will deposit polymer at different thicknesses. At the relatively high speed of 20 µm/s, only a single monolayer is deposited as shown by the line average to the right of the image. Lower speeds deposit thicker polymer lines as shown by line averages in (c).

**Figure 3:**
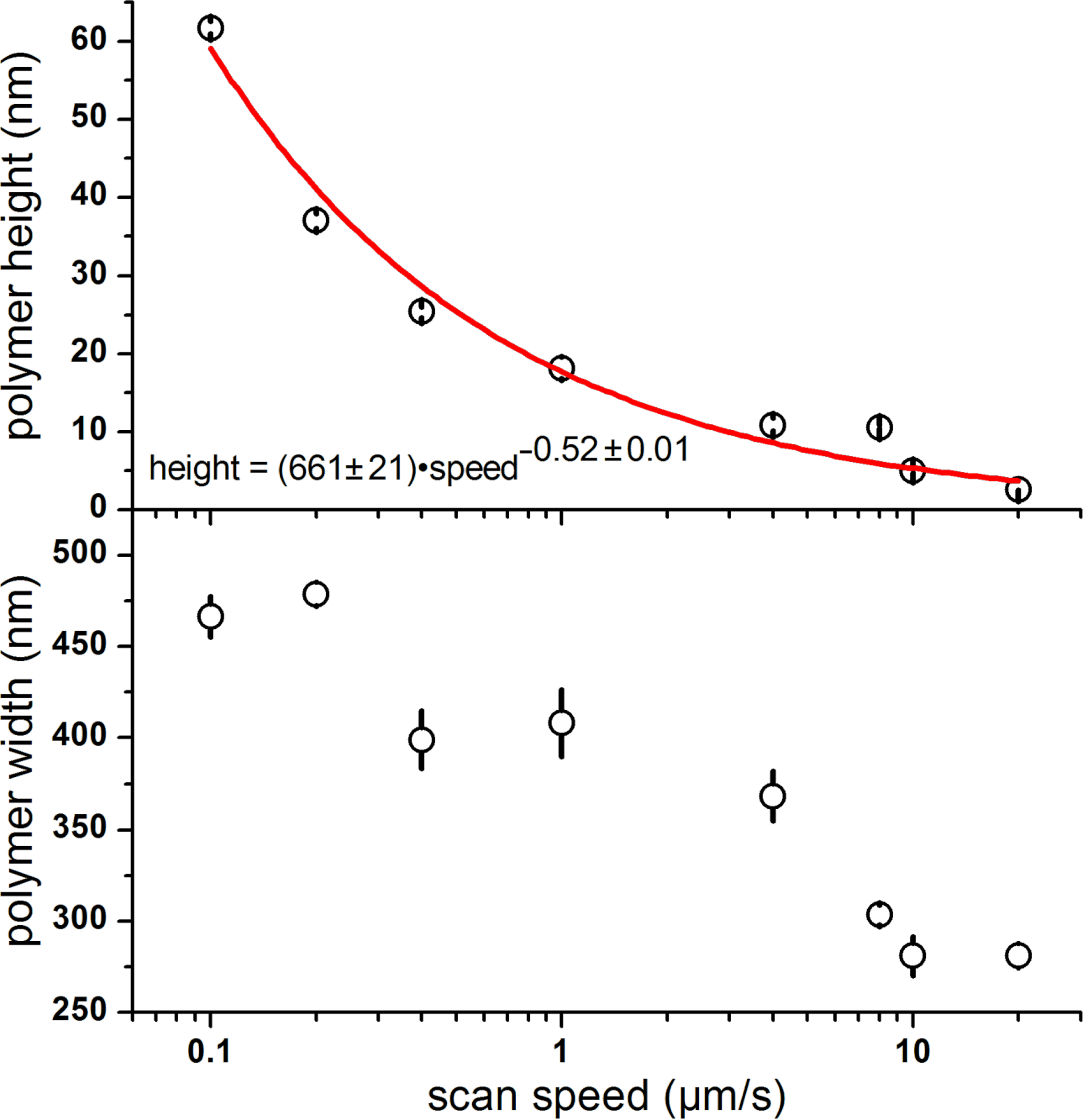
The polymer deposit heights and widths of PDDT deposited onto Si substrate (non-UHV prepared) as a function of scanning speed. Both the height and width decrease monotonically with tip speed.

While heating the probe to the vacuum melting temperature of the PDDT, the tip was rasterized across the “as is” native oxide Si substrate at different speeds. We found that monolayer-by-monolayer control of the film thickness, as previously established under nitrogen, is also possible under UHV. Figure 2 shows two polymer nanowire lines written at different speeds. Assuming a thickness of 2.6 nm for each PDDT monolayer as previously determined by XRD [14], the polymer deposited by the probe moving at 20 µm/s was only a single monolayer thick, with the structure written at 8 µm/s being four monolayers thick. The widths of the deposited polymer structures were 280 nm at 20 µm/s and 303 nm at 8 µm/s, with the width principally determined by the relatively blunt silicon tip. Note that recent advances – where the tips remain sharp due to a coating of wear-resistant diamond – readily show line thicknesses of 40 nm [17]. The line width and heights were measured as a function of the probe speed (Figure 3). The heights of the deposited polymer structures roughly decrease as the inverse square root of the scan speed. The widths of the deposited structures decrease monotonically with the scan speed but do not show a clear power law relationship. When patterning under ambient conditions, dimensional control may be achieved by varying the tip temperature; however, the tip temperature was fixed in UHV to limit the number of experimental parameters.

**Figure 4:**
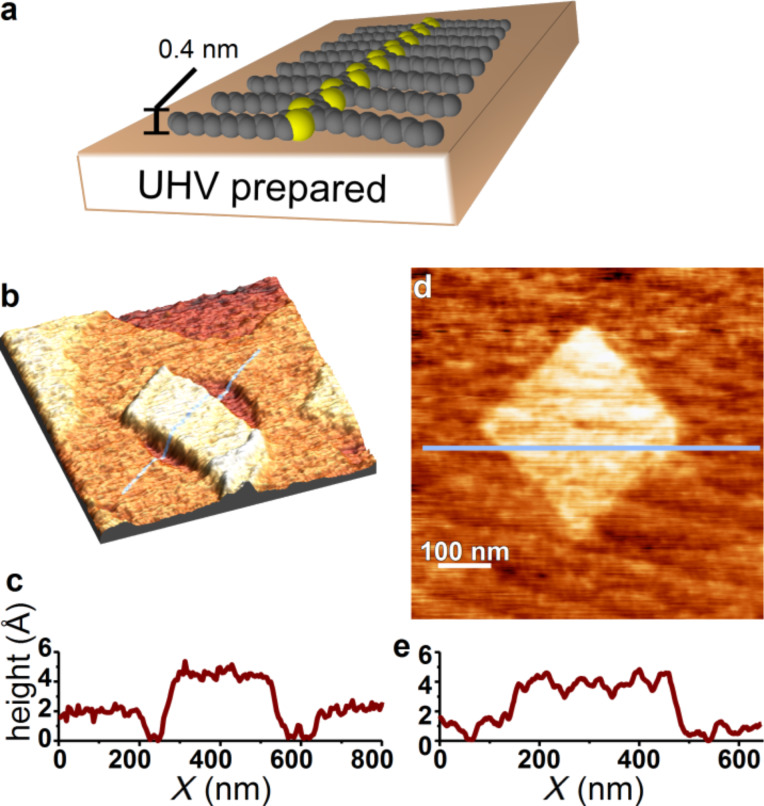
(a) Deposition onto the UHV prepared Si substrate in UHV shows the polymer lying on its side. (b) Polymer deposited across a Si step edge an atom thick. (c) The cross section [pale blue line in (b)] shows that the polymer thickness is 0.4 nm, indicating that the polymer molecules are lying flat. (d) A second image of polymer deposited on a UHV-prepared clean silicon surface with diagonal monatomic steps that go from the upper left to the lower right. (e) Cross section from (d) that again gives a polymer thickness of 0.4 nm.

Polymer nanostructures were also written on atomically clean and flat Si(001)-2×1 (Figure 4) where monoatomic steps are clearly visible. Interestingly, we found that surface chemistry of the silicon substrate had a major effect on the apparent structure of the deposited polymer as determined by the monolayer film thickness. On the native oxide surface, PDDT self-assembles in such way that the side chains are perpendicular to the surface (Figure 2a), as typically observed for PDDT deposited on non-UHV prepared surfaces under ambient conditions [18]. The upright orientation is due to the hydrophobic alkyl side chains minimizing their exposure to the hydrophilic oxide substrate. In contrast, PDDT written on Si(001)-2×1 has a film thickness of ~0.4 nm, corresponding to polymer side-chains oriented parallel to the surface, as illustrated in Figure 4a. Note that the thickness of our films lies intermediate to values reported previously for PDDT on other substrates. Scifo et al. used STM to measure the thickness in UHV of a PDDT film drop cast on highly oriented pyrolytic graphite (HOPG) and reported a film thickness of 0.24 ± 0.04 nm [19]. In contrast, Terada et al. [20] reported poly(3-hexylthiophene) (P3HT) on H-terminated Si(100) in UHV to be 0.5 nm thick. Our measured value is closer to the 0.4 nm intermolecular spacing measured for thick films of PDDT [14]. In the prior STM measurements, the measured thickness is a convolution of the topographic height and electronic properties of the polymer film, complicating the comparison. However, the polymer’s lying flat strongly suggests that alkyl side chains must interact more favorably with the silicon surface than with the oxide surface and so has a significant impact on the observed molecular film thickness.

## Conclusion

In conclusion, we have developed a method for direct, additive deposition of polymer in UHV using thermal dip-pen nanolithography. The molecular structure of the written PDDT monolayer nanostructure films depends on the chemistry of the silicon surface. Oxide termination leads to polymer side chains aligning perpendicular to the substrate, whereas silicon termination leads to the polymer lying flat. The thickness of the deposited polymer is a function of the speed of the scanning probe and may be controlled monolayer-by-monolayer. This new UHV-compatible direct-write technique should be of value both for nanoscale lithography of polymer structures and for the study of molecularly-ordered polymer nanostructures. This result would also open a new method of studying polymer-semiconductor surface interaction at a molecular level which is useful to develop polymer-based electronics compatible with inorganic semiconductor technology.

## Experimental

The silicon wafer substrates were prepared using one of two protocols. In both protocols, substrates for depositing PDDT were scribed from Sb-doped Si(001) wafers (0.01 to 0.02 Ω·cm) oriented to within 0.1° of (001). The substrates were then sonicated in CHCl_3_, dried with a stream of N_2,_ and transferred into the UHV chamber (base pressure ~5 × 10^−11^ Torr). In the first protocol, the substrate was used as-is to take advantage of the ~2 nm thick native oxide. In the second cleaning protocol, samples were prepared to leave an atomically pristine, 2×1-reconstucted Si(001) surface. In this protocol, the substrates were initially degassed in UHV overnight at 500 °C and resistively heated for 30 s at 1230 °C, cooled down for at least 5 min, and then briefly heated again to 1230 °C for 5 s, while maintaining a pressure below 1 × 10^−9^ Torr. Depending on the sample holder history, several heating-cycles were necessary before the pressure could be maintained below 1 × 10^−9^ Torr.

Poly(3-dodecylthiophene) (PDDT) was purchased from Sigma-Aldrich (*M*_w_ ~ 60,000) and used without any further purification. To pattern PDDT via tDPN in UHV, the heatable cantilever was first mounted on a UHV tip holder. Next, a solution of 0.1% by volume of PDDT in chloroform was loaded onto the cantilever and tip by using a 3 mm diameter loop of copper wire containing the solution in the meniscus. Using a micromanipulator, the tip was immersed into the droplet, dried on a hot plate at 60 °C and then loaded into the UHV chamber.

## References

[1] Wolf S, Tauber R (1986). Silicon Processing for the VLSI Era.

[2] de Gans B-J, Duineveld P C, Schubert U S (2004). Adv Mater.

[3] Ginger D S, Zhang H, Mirkin C A (2004). Angew Chem, Int Ed.

[4] Ruiz S A, Chen C S (2007). Soft Matter.

[5] Lee W-K, Sheehan P E (2008). Scanning.

[6] Tseng A A, Notargiacomo A, Chen T P (2005). J Vac Sci Technol, B: Microelectron Nanometer Struct–Process, Meas, Phenom.

[7] Piner R D, Zhu J, Xu F, Hong S, Mirkin C A (1999). Science.

[8] Jaschke M, Butt H-J (1995). Langmuir.

[9] Sheehan P E, Whitman L J (2002). Phys Rev Lett.

[10] Sheehan P E, Whitman L J, King W P, Nelson B A (2004). Appl Phys Lett.

[11] Nelson B A, King W P, Laracuente A R, Sheehan P E, Whitman L J (2006). Appl Phys Lett.

[12] Lee W K, Dai Z, King W P, Sheehan P E (2010). Nano Lett.

[13] Roncali J (1992). Chem Rev.

[14] Prosa T J, Winokur M J, Moulton J, Smith P, Heeger A J (1992). Macromolecules.

[15] Laracuente A R, Yang M, Lee W K, Senapati L, Baldwin J W, Sheehan P E, King W P, Erwin S C, Whitman L J (2010). J Appl Phys.

[16] Lee J, Beechem T, Wright T L, Nelson B A, Graham S, King W P (2006). J Microelectromech Syst.

[17] Fletcher P C, Felts J R, Dai Z, Jacobs T D, Zeng H, Lee W, Sheehan P E, Carlisle J A, Carpick R W, King W P (2010). ACS Nano.

[18] Yang M, Sheehan P E, King W P, Whitman L J (2006). J Am Chem Soc.

[19] Scifo L, Dubois M, Brun M, Rannou P, Latil S, Rubio A, Grévin B (2006). Nano Lett.

[20] Terada Y, Miki K, Fujimori M, Heike S, Suwa Y, Hashizume T (2005). J Appl Phys.

